# Editorial: From Structure to Signalsomes: New Perspectives About Membrane Receptors and Channels

**DOI:** 10.3389/fpls.2019.00682

**Published:** 2019-05-22

**Authors:** Yi Ma, Kai He, Gerald A. Berkowitz

**Affiliations:** ^1^Plant Science, University of Connecticut, Mansfield, CT, United States; ^2^School of Life Sciences, Lanzhou University, Lanzhou, China

**Keywords:** ion channels, signalosome, stress response and growth, signal transduction, membrane receptors

The plasma membrane (PM) forms a selective barrier between the interior of the cell and the external environment. The PM structure and constituents can act as a sentry to facilitate perception of changes in the environment to trigger intricate signaling cascades, leading to fine-tuned adaptive responses. The PM contains a plethora of proteins that are channels, transporters, receptors or enzymes. These proteins are involved in a number of cell activities such as the following: (a) transport of molecules or other substances across the plasma membrane, (b) perception of external environmental cues that cause perturbations in plant homeostasis (for example, temperature stress and salinity), and (c) patterning the cellular response to internal (plant) signal molecules such as hormones or peptides. In these cases of PM function, the transduction of signal perception orchestrates downstream events in the cytosol that modify cell function to respond to the signal (Palme, 2012). The proteins could function together as a signalosome in a discreet functional unit (a plasma membrane “nanodomain”) to facilitate the signal transduction processes that are responsible for plant growth and development or defense responses (Tapken and Murphy, 2015).

This Frontiers Research Topic have compiled a collection of cutting-edge research articles and up to date reviews on the study of plasma membrane proteins that are involved in abiotic or biotic stress, cell growth and plant development. The article collection that composes this Research Topic includes 13 original research articles, one review and one perspective. Contributions were made by 115 authors from Australia, China, Denmark, Italy, Tunisia, United Arab Emirates, and the United States.

Regulation of plasma membrane transporters is crucial for ion homeostasis in plants under a variety of abiotic stress conditions. Hazzouri et al. identified the *HKT1;5* gene and its associated QTL in barley. This work provides new insights on the breeding of salt tolerant barley. Among the small grains, this crop is often grown in soil with saline stress. Another HKT member in rice, OsHKT2;4, was shown to be a low-affinity Mg^2+^ transporter that mediates Mg^2+^ homeostasis under high Mg^2+^ conditions (Zhang et al.). In addition, Yan et al. demonstrated that Mg^2+^ transporters MGT6 and MGT7 has additive dual functions in plant adaptation to a low Mg^2+^ environment and detoxification of excess Mg^2+^ under high Mg^2+^ conditions. Niu et al. showed that the lipid flippase activity of the P_4_-type ATPase ALA6 plays a critical role for membrane stability under heat stress. Yu et al. demonstrated that overexpression of poplar ABA receptors *PtPYRL1* or *PtPYRL5* significantly enhanced cold, drought, and osmotic tolerance in poplar trees.

Cyclic nucleotide-gated ion channels (CNGCs) and Ca^2+^ have been shown to play vital roles in plant defense responses to various pathogens (Ma and Berkowitz, 2011; Moeder et al., 2011). Zhang et al. showed that silencing of tomato *CNGC1* and *CNGC14* enhanced *Xanthomonas oryzae* pv. *oryzicola* (*Xoc*) induced hypersensitive responses and non-host resistance to *Xoc*, suggesting a negative regulatory role of these CNGCs in non-host resistance and basal immune responses (also referred to as pathogen associated molecular pattern (PAMP)-triggered immunity). Studies have revealed contradictory roles of CNGCs in pathogen resistance; clearly this ion channel-mediated signal transduction system responding to pathogen perception should be the focus of further studies in the future to better understand how Ca^2+^ and CNGCs regulate plant immune defense responses to microbial pathogens.

Plasma membrane localized transporters and receptors regulate hormone mediated cell elongation. Among the transporters facilitating this signaling are the PIN-FORMED (PIN) family of auxin transporters. Ni et al. showed that *S*-nitrosoglutathione-regulated PIN2 endocytosis is important for root elongation. Some members of the large family of leucine-rich repeat receptor-like kinases (LRR-RLKs) act as receptors for hormones and peptides; in some cases these receptors can transduce developmental signals into altered cell growth. Among these LRR-RLKs involved in facilitating peptide signals and modulating growth is ERECTA. Qu et al. demonstrated that ERECTA regulates hypocotyl elongation by activating auxin biosynthesis. In another study of ERECTA, in this case focusing on shade avoidance, Du et al. showed that under shade, ERECTA-regulated hypocotyl elongation is controlled by both auxin and gibberellins.

Yang et al. and Li et al. employed transcriptomic approaches to reveal genes involved in Ca^2+^ regulated peanut pod development, which could also be regulated by hormonal signaling. Ca^2+^ signaling is involved as a secondary cytosolic messenger responding to ligand perception by cell membrane receptors in myriad signal transduction pathways, including those responding to pathogen perception during defense response, and in response to hormone/peptide cues patterning growth and development. One such signaling pathway that impacts cell expansion involves the tyrosine-sulfated pentapeptide phytosulfokine (PSK) acting as a peptide hormone signal that activates cell responses upon binding to its cognate LRR-RLK receptor PSKR1. Irving et al. focused on the signaling pathway activated by PSKR1 perception of the PSK ligand in a review of a subset of LRR-RLK cell membrane receptors. Irving et al. noted that, in addition to having cytosol kinase domains that facilitate downstream signaling, PSKR1 (and similar LRR-RLKs) also facilitate downstream signaling involving cGMP generation and CNGC activation which then leads to cytosolic Ca^2+^ elevation. Irving et al. provide some insightful context for signaling in presenting the PSK/PSKR1 interaction as impacting other membrane proteins beyond CNGCs. They point out that PSKR1 is thought to function as a complex with other proteins including the coreceptor BAK1, a CNGC, and the cell membrane proton pumps AHA1 and AHA2. These proteins might act as a unit, or signalosome in a nanodomain at the cell surface.

A peptide hormone/LRR-RLK cell membrane receptor signaling pathway similar to PSK/PSKR1 involves another tyrosine-sulfated peptide, PSY1, and its cognate receptor PSY1R. Oehlenschlæger et al. studied PSY1-dependent transphosphorylation of residues in the activation loop of PSY1R, which are critical for the activation of the receptor and downstream signaling. They demonstrated that PSY1R activation involves interaction (transphosphorylation) with the BAK1 coreceptor (and other members of the BAK1 membrane protein family) in a fashion similar to PSKR1. Intriguingly, signaling by the PSKR1 and PSY1R receptors also involves activation of a cell membrane proton pump [in this case, only AHA1; (Fuglsang et al., 2014)], and both receptors are involved in cell expansion activated during developmental programs in plants. The two pathways downstream from these receptors are also involved in signaling that leads to plant defense against pathogen invasion (Mosher and Kemmerling, 2013). Gao et al. studied another aspect of the coreceptor BAK1 involvement in immune signaling. In their study, they found that some signal associated with light interacts with programmed cell death; one possible downstream outcome from immune signaling. The immune responses involving BAK1 as an upstream component were found to involve proteins of the salicylic acid signaling pathway.

Gehring and Turek summarized the current progress of work on cyclic nucleotide monophosphates (cAMP and cGMP), which activate CNGCs. And, as mentioned above, Irving et al. provided a review and perspectives on the research of cyclic nucleotides using cGMP and PSKR1 as an example. Although the presence of plant nucleotide cyclases that synthesize cAMP and cGMP in plant cells is still controversial, more and more evidence supports their roles in numerous signal transduction pathways in plant cells; the focus of some of the contributions to this Research Topic.

More than 80% of the global population live in poverty; most of this problem can be attributed to adverse agricultural conditions such as salinity, drought, lack of nutrition, temperature stress or pathogen infection. The studies included in this Research Topic could provide new insights on how to solve or ameliorate such problems through the modifications of traits controlled by certain genes.

## Author Contributions

YM wrote the editorial article. GB edited and modified the article. KH proofread the article.

### Conflict of Interest Statement

The authors declare that the research was conducted in the absence of any commercial or financial relationships that could be construed as a potential conflict of interest.
